# Integration of the GRIm Score with Pathologic Immune and Stromal Markers to Develop a Combined Prognostic Model in Gastric Cancer: A Retrospective Single-Center Study

**DOI:** 10.3390/medicina62010192

**Published:** 2026-01-16

**Authors:** Gökhan Öztürk, Ebru Taştekin, Canberk Topuz, Aysun Fatma Akkuş, Tayyip İlker Aydın, Sernaz Topaloğlu, Bülent Erdoğan, Muhammet Bekir Hacıoğlu, Ahmet Küçükarda

**Affiliations:** 1 Department of Medical Oncology, Trakya University Faculty of Medicine, 22030 Edirne, Türkiye; aysunfatmadogan@gmail.com (A.F.A.); ilker6125@gmail.com (T.İ.A.); sernaz.uzunoglu@gmail.com (S.T.); berdoga@hotmail.com (B.E.); mbekirhacioglu@yahoo.com (M.B.H.); ahmetkucukarda22@gmail.com (A.K.); 2 Department of Pathology, Trakya University Faculty of Medicine, 22030 Edirne, Türkiye; ebrutastekin@hotmail.com (E.T.); cnbrktpz@gmail.com (C.T.)

**Keywords:** gastric cancer, GRIm score, tumor microenvironment, programmed death-ligand 1 (PD-L1), tumor-stroma ratio (TSR), tumor-infiltrating lymphocytes (TIL), prognostic biomarker, systemic inflammation

## Abstract

*Background and Objectives*: The Gustave Roussy Immune (GRIm) score, reflecting systemic inflammation and nutritional status, has emerged as a simple and reproducible prognostic biomarker in various malignancies. However, its prognostic interaction with tumor microenvironmental factors remains unclear in gastric cancer. The primary aim of this study was to evaluate the prognostic value of the GRIm score in patients with resectable gastric adenocarcinoma, while the secondary aim was to determine whether integrating the GRIm score with tumor microenvironment–related pathological markers could improve prognostic stratification. *Materials and Methods*: This retrospective study analyzed 188 patients with resectable gastric adenocarcinoma treated at the Trakya University Faculty of Medicine between 2007 and 2018. GRIm scores were calculated from preoperative lactate dehydrogenase (LDH), albumin, and neutrophil-to-lymphocyte ratio (NLR) values. Pathologic parameters, including programmed death-ligand 1 (PD-L1) expression (combined positive score [CPS] ≥ 1 vs. <1), tumor–stroma ratio (TSR; stromal component ≥ 50% vs. <50%), and tumor-infiltrating lymphocyte (TIL) density (CD8+ ≥ 10% vs. <10%), were evaluated on surgical specimens. Survival outcomes were assessed using Kaplan–Meier and multivariate Cox analyses. *Results*: The study population had a mean age of 61.8 years and was predominantly male (72.3%). Patients with low GRIm scores had significantly longer disease-free survival (DFS; 24 vs. 12 months; *p* = 0.004) and overall survival (OS; 32 vs. 19 months; *p* = 0.006). In multivariate analysis, the GRIm score remained an independent predictor for both disease-free survival (*p* = 0.035) and overall survival (*p* = 0.044). Among combined models, the GRIm–TSR classification provided the most pronounced stratification (median DFS = 35 vs. 12 months; OS = 45 vs. 19 months; *p* = 0.014 and 0.001, respectively), retaining independent prognostic significance (hazard ratio [HR] = 1.23; *p* = 0.005). Integrating GRIm with PD-L1 and TIL density also improved prognostic discrimination. *Conclusions*: The GRIm score is a robust and cost-effective biomarker that independently predicts disease-free survival and overall survival in resectable gastric adenocarcinoma. Its combination with microenvironmental markers—PD-L1, TIL, and TSR—captures complementary biological dimensions of tumor aggressiveness, offering an integrative and clinically feasible framework for individualized risk assessment and postoperative management. Prospective multicenter validation is warranted.

## 1. Introduction

Gastric cancer remains a significant global health concern and is reported to be the fifth most frequently diagnosed malignancy and the fourth leading cause of cancer-related deaths worldwide [1,2,3]. According to the most recent GLOBOCAN 2024 data, approximately 1.1 million new cases are diagnosed each year, and nearly 770,000 deaths are attributed to gastric cancer [1,2]. Despite advances in screening programs, surgical techniques, and systemic therapies, the majority of patients are still diagnosed at an advanced stage, and only about 25–30% are identified at a localized stage amenable to curative surgery [4,5]. In contrast, approximately 40% of cases present with distant metastases at diagnosis, which contributes to a global five-year overall survival rate of less than 30% [5,6]. These data indicate that gastric cancer continues to carry a high mortality burden and underscore the critical importance of early detection and the development of reliable prognostic biomarkers for optimizing clinical management [3,6,7].

Nutritional status and systemic inflammation are well recognized as key determinants of cancer prognosis [8]. Based on this biological rationale, the Gustave Roussy Immune Score (GRIm-Score) was developed as a prognostic biomarker that simultaneously reflects both inflammatory and nutritional conditions. This scoring system, which incorporates the neutrophil-to-lymphocyte ratio (NLR), lactate dehydrogenase (LDH), and albumin (ALB) levels, was originally designed to assist in patient selection for immunotherapy clinical trials and demonstrated superior performance in predicting prognosis [9]. Subsequent studies confirmed the prognostic value of the GRIm-Score across various malignancies, including non-small cell lung cancer [10,11].

The tumor microenvironment (TME) is a dynamic and complex network comprising interactions between cancer cells and stromal and immune components, playing a pivotal role in tumor progression and therapeutic response [12,13,14,15]. In recent years, the expression of programmed death-ligand 1 (PD-L1) has been identified as both a prognostic and predictive biomarker, particularly with significant clinical relevance for predicting response to immunotherapy [16]. Moreover, tumor-infiltrating lymphocytes (TILs) represent immune cell populations that migrate into tumor tissue, and multiple studies have demonstrated that TIL density is significantly associated with survival in numerous solid tumors, including gastric cancer, thus establishing TILs as an important prognostic factor [17]. In addition, the tumor–stroma ratio (TSR), which reflects the proportion of stromal components within tumor tissue, has been defined as an independent factor influencing tumor biology and patient outcomes [18,19,20,21,22]. Studies in gastric cancer have shown that a low TSR, characterized by high stromal content, correlates with poorer survival outcomes, suggesting that TSR may serve as a valuable prognostic parameter representing the structural and functional features of the tumor microenvironment [18].

Gastric cancer remains a biologically heterogeneous malignancy with persistently high mortality rates [3,6]. Therefore, identifying reliable prognostic parameters is of critical importance for predicting the clinical course of the disease and for individualizing therapeutic strategies [5]. 

Most studies in the existing literature have evaluated clinical or pathological variables independently, while prognostic models integrating both domains remain extremely limited.

However, approaches that consider these parameters in a comprehensive manner have demonstrated markedly stronger predictive performance for patient outcomes.

Based on this unmet need, the present study aimed to develop a more reliable and clinically meaningful prognostic prediction model by combining clinical and pathological indicators in patients with resectable gastric cancer.

## 2. Materials and Methods

### 2.1. Patient Selection

This retrospective study included patients diagnosed with gastric adenocarcinoma and followed at the Department of Medical Oncology, Trakya University Faculty of Medicine (Edirne, Türkiye).

Only patients aged 18 years or older who underwent subtotal or total gastrectomy with curative intent between January 2008 and October 2018 and for whom complete clinicopathologic, laboratory, and follow-up data were available were included in the analysis.

Patients diagnosed outside the predefined study period were excluded due to insufficient follow-up data for reliable survival analysis. In addition, cases with recurrent disease, distant metastasis, or prior neoadjuvant chemotherapy, histologically confirmed second primary malignancies, or missing survival or follow-up data were excluded to avoid treatment-related morphological alterations and potential prognostic bias.

Trakya University Faculty of Medicine is a tertiary referral center where gastric cancer surgery is routinely performed within a multidisciplinary framework by experienced surgical oncology teams.

The study protocol was approved by the Trakya University Faculty of Medicine Scientific Research Ethics Committee (Protocol Code: TUTF-GOBAEK 2025/406) on 06 October 2025.

### 2.2. Definition of the GRIm Score

For each patient, the Gustave Roussy Immune (GRIm) score was calculated based on three routine laboratory parameters: serum lactate dehydrogenase (LDH), serum albumin level, and the neutrophil-to-lymphocyte ratio (NLR).

The following cut-off values were used: LDH > upper limit of normal (ULN), albumin < 35 g/L, and NLR > 6.

Each abnormal parameter was assigned 1 point, yielding a total score ranging from 0 to 3.

Patients were categorized into two prognostic groups:

- Low GRIm score: 0–1 points.
- High GRIm score: 2–3 points.

The GRIm score was originally developed as a prognostic biomarker reflecting systemic inflammation and nutritional status in patients with metastatic cancer. In the present study, the GRIm score was analyzed in relation to pathologic parameters (TSR, TIL, PD-L1) and survival outcomes.

### 2.3. Pathologic Parameters

Data on PD-L1 expression, tumor–stroma ratio (TSR), and tumor-infiltrating lymphocytes (TIL, evaluated as peritumoral lymphocytes [PTLs]) were obtained from a previously conducted, ethics-approved study performed at the Department of Pathology, Trakya University Faculty of Medicine (Protocol Code: TUTF-GOBAEK 2023/72).

In that study, PD-L1 expression was evaluated using the DAKO 22C3 clone on the Ventana Benchmark Ultra automated immunohistochemistry platform, TIL density was assessed by CD8/144b immunostaining, and TSR was determined morphologically on hematoxylin–eosin (H&E) stained slides. In the present study, TSR was defined as the proportion of stromal area within the tumor tissue.

All immunohistochemical and histopathologic evaluations were performed in accordance with institutional standard protocols. In the current analysis, these pathological data were combined with the retrospectively calculated GRIm scores for prognostic assessment.

### 2.4. Combined Grouping and Prognostic Stratification

To investigate the prognostic interactions between systemic and tumor microenvironmental factors, patients were further stratified into combined subgroups based on the GRIm score and selected pathological parameters, including programmed death-ligand 1 (PD-L1), tumor–stroma ratio (TSR), and tumor-infiltrating lymphocytes (TIL).

Four separate survival analyses were performed:

(1) GRIm score alone (low vs. high; cut-off: LDH > ULN, albumin < 35 g/L, NLR > 6),
(2) combined analysis of GRIm score and PD-L1 expression (cut-off: combined positive score [CPS] ≥ 1),
(3) combined analysis of GRIm score and tumor–stroma ratio (TSR) (cut-off: >50% stroma considered stroma-high),
(4) combined analysis of GRIm score and tumor-infiltrating lymphocytes (TIL) (cut-off: ≥10% CD8-positive TIL density defined as high).

For each combined model, patients were classified into four prognostic subgroups according to the intersection of GRIm score (low/high) and the corresponding pathological parameter (positive/negative or low/high).

These composite groups were compared to assess survival differences using Kaplan–Meier–based analyses. This stratification allowed the evaluation of how systemic inflammatory and nutritional status (as reflected by the GRIm score) interacts with tumor microenvironmental immune and stromal features (PD-L1 expression, TSR, and TIL density) in determining clinical outcomes. All combined models were subsequently tested in multivariate Cox regression to determine their independent prognostic significance alongside conventional clinicopathologic variables.

### 2.5. Statistical Analysis

All statistical analyses were conducted using IBM SPSS Statistics, version 25.0 (IBM Corp., Armonk, NY, USA). Continuous variables are presented as median (interquartile range, IQR) or mean ± standard deviation (SD), whereas categorical variables are summarized as counts and percentages. Between-group comparisons of clinicopathologic characteristics were performed using the Chi-square or Fisher’s exact test for categorical variables, and the Student’s *t*-test or Mann–Whitney U test for continuous variables, as appropriate.

Overall survival (OS) and, when applicable, disease-free survival (DFS) were estimated by the Kaplan–Meier method, and survival differences between subgroups were compared using the log-rank test. Variables with a *p* value < 0.10 in univariate analyses were entered into multivariate Cox proportional-hazards regression models to identify independent prognostic factors. Results were expressed as hazard ratios (HRs) with 95% confidence intervals (CIs).

A two-sided *p* value < 0.05 was considered statistically significant.

Descriptive and comparative analyses were performed for the GRIm score (low vs. high) as well as for combined subgroup models integrating the GRIm score with PD-L1 expression (CPS ≥ 1), tumor–stroma ratio (TSR > 50%), and tumor-infiltrating lymphocytes (TIL ≥ 10%). These combined models were further assessed in multivariate Cox analyses to determine their independent prognostic significance.

As this was a retrospective exploratory prognostic study, no formal a priori sample size calculation or statistical test was performed to determine a minimum required number of patients. All consecutive eligible patients during the study period were included, and the adequacy of the sample size was evaluated based on the number of observed events and the stability of effect estimates in multivariate Cox regression analyses.

## 3. Results

### 3.1. Baseline Characteristics

A total of 188 patients diagnosed with gastric adenocarcinoma were included in the study.

The mean age at diagnosis was 61.8 ± 11.5 years (median: 62; range: 28–87), and the majority were male (72.3%, n = 136). Histologically, tubular adenocarcinoma was the most frequent subtype (45.2%, n = 85), followed by poorly cohesive carcinoma (22.3%, n = 42), mixed (20.2%, n = 38), papillary (6.4%, n = 12), mucinous (3.7%, n = 7), and micropapillary (2.1%, n = 4). Lymphovascular invasion was present in 85.1% (n = 160), and perineural invasion in 66.0% (n = 124) of cases.

Lymph node involvement was categorized as N0 (23.9%, n = 45), N1 (25.5%, n = 48), N2 (14.9%, n = 28), and N3 (35.6%, n = 67). According to T stage, T4 was the most common (54.8%, n = 103), followed by T3 (26.6%, n = 50). Tumors were most frequently located in the corpus (28.7%, n = 54) and antrum (26.1%, n = 49), followed by diffuse-type (23.9%, n = 45), cardia (14.4%, n = 27), fundus (4.3%, n = 8), and pylorus (2.7%, n = 5). Of the entire cohort, 181 patients (96.3%) with T2 or higher tumor stage and/or lymph node positivity received adjuvant doublet chemotherapy with 5-fluorouracil plus oxaliplatin.

A low tumor–stroma ratio (TSR < 50%) was observed in 56.9% (n = 107) of cases, whereas high TSR (≥50%) was found in 43.1% (n = 81). Regarding immunohistochemical markers, PD-L1 CPS ≥ 1 was positive in 49.5% (n = 93) and negative in 50.5% (n = 95).

When a higher threshold was applied (PD-L1 CPS ≥ 10), 20% (n = 37) were positive.

TIL positivity ≥10% was detected in 73.4% (n = 138). (Detailed baseline clinicopathological features are summarized in Table 1.)

### 3.2. Prognostic Impact of the GRIm Score

When patients were stratified into low (0–1) and high (2–3) GRIm score categories, the distribution of most baseline clinicopathologic characteristics was balanced between the groups. No significant differences were observed in age, sex, tumor location, histologic subtype, or grade. Although minor imbalances in lymph node involvement and lymphovascular invasion were noted, they did not reach statistical significance in univariate analysis. This indicates that GRIm score categories were largely independent of major clinicopathologic variables.

**Figure 1:**
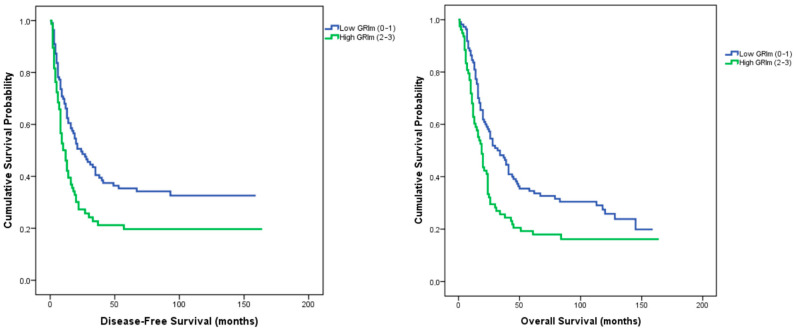
Kaplan–Meier curves for disease-free survival and overall survival according to GRIm scores.

In survival analyses, the GRIm score was significantly associated with both disease-free survival (DFS) and overall survival (OS). Median DFS was 24 months (95% CI: 13.6–34.3) in the low GRIm group versus 12 months (95% CI: 8.1–15.8) in the high GRIm group (log-rank *p* = 0.004). Similarly, median OS was 32 months (95% CI: 21.7–42.3) in the low GRIm group and 19 months (95% CI: 15.2–22.8) in the high GRIm group (log-rank *p* = 0.006) (Figure 1).

In Cox regression analyses, higher T and N stages, perineural invasion, and a high GRIm score were significantly associated with unfavorable survival outcomes. In multivariate models, the GRIm category remained an independent prognostic factor for both disease-free survival (HR = 1.45, 95% CI 1.01–2.09, *p* = 0.045) (Table 2) and overall survival (HR = 1.41, 95% CI 1.01–1.97, *p* = 0.044) (Table 3), together with advanced T and N stages. These findings indicate that the GRIm score, reflecting systemic inflammatory and nutritional status, independently predicts both recurrence risk and long-term mortality in patients with resected gastric adenocarcinoma. As an exploratory assessment of model discrimination, the Harrell’s concordance index (C-index) for disease-free survival was 0.62.

### 3.3. Combined Analysis of GRIm Score and PD-L1 Expression

Patients were stratified into four subgroups based on GRIm score and PD-L1 CPS ≥1 status: low GRIm/PD-L1–negative, low GRIm/PD-L1–positive, high GRIm/PD-L1–negative, and high GRIm/PD-L1–positive. The distribution of clinicopathologic characteristics, including T and N stage, histologic subtype, tumor location, grade, and lymphovascular invasion, showed no significant differences among the four groups (all *p* > 0.05).

**Figure 2:**
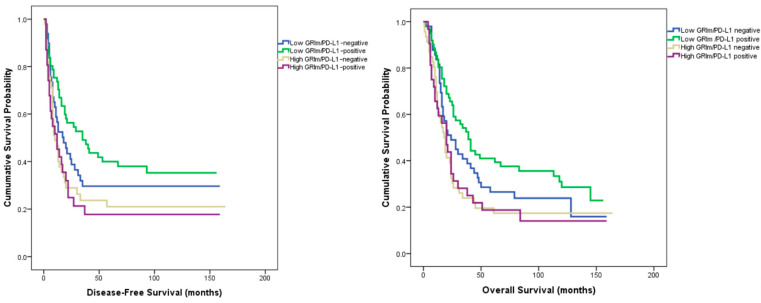
Kaplan–Meier curves for disease-free and overall survival according to the combined GRIm–PD-L1 classification.

Regarding disease-free survival, median survival times were 18.0 months (95% CI: 8.4–27.6) in the low GRIm/PD-L1–negative group, 35.0 months (95% CI: 14.7–55.2) in the low GRIm/PD-L1–positive group, 10.0 months (95% CI: 5.9–14.1) in the high GRIm/PD-L1–negative group, and 12.0 months (95% CI: 4.4–19.6) in the high GRIm/PD-L1–positive group (log-rank χ^2^ = 9.416, *p* = 0.024). For overall survival, the corresponding medians were 24.0 months (95% CI: 12.6–35.4), 39.0 months (95% CI: 29.2–48.8), 18.0 months (95% CI: 13.6–22.4), and 20.0 months (95% CI: 13.1–26.9) for the same respective subgroups (log-rank χ^2^ = 9.021, *p* = 0.029) (Figure 2).

After adjustment for conventional clinicopathologic variables in multivariate Cox regression analysis, the combined GRIm–PD-L1 classification remained significantly associated with disease-free survival (HR = 1.191, 95% CI = 1.004–1.413, *p* = 0.045) and showed a marginal association with overall survival (HR = 1.166, 95% CI = 0.995–1.367, *p* = 0.057).

### 3.4. Combined Analysis of GRIm Score and Tumor–Stroma Ratio (TSR)

In the combined analysis of GRIm score and tumor–stroma ratio (TSR), patients were stratified into four subgroups: low GRIm/low TSR, low GRIm/high TSR, high GRIm/low TSR, and high GRIm/high TSR.

The distribution of clinicopathologic characteristics, including tumor location, histologic grade, lymphovascular and lymphatic invasion, and sex, did not differ significantly among the four subgroups (all *p* > 0.05).

However, significant intergroup differences were observed in T stage (*p* = 0.032), lymph node stage (*p* = 0.042), perineural invasion (*p* < 0.001), and histologic subtype (*p* = 0.012).

Median disease-free survival (DFS) was 35 months in the low GRIm/low TSR group, 20 months in the low GRIm/high TSR group, 8 months in the high GRIm/low TSR group, and 12 months in the high GRIm/high TSR group (log-rank χ^2^ = 10.694, *p* = 0.014).

**Figure 3:**
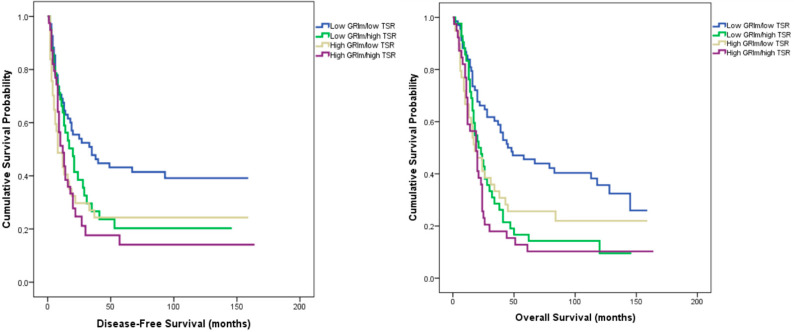
Kaplan–Meier curves for disease-free survival (**left**) and overall survival (**right**) according to combined GRIm–TSR classification.

The corresponding overall survival (OS) values were 45, 21, 18, and 19 months, respectively (log-rank χ^2^ = 17.143, *p* = 0.001) (Figure 3).

In multivariate Cox regression analysis adjusting for conventional prognostic parameters (T and N stage, grade, perineural and lymphovascular invasion, PD-L1 status, and TIL percentage), the combined GRIm–TSR classification remained independently associated with both DFS and OS.

The GRIm–TSR groups showed a significant effect on recurrence risk (HR = 1.18, 95% CI 1.00–1.37, *p* = 0.044) and overall mortality (HR = 1.23, 95% CI 1.06–1.42, *p* = 0.005).

### 3.5. Combined Analysis of GRIm Score and Tumor-Infiltrating Lymphocytes (TIL)

In the combined GRIm–TIL classification, patients were categorized into four subgroups: low GRIm/low TIL, low GRIm/high TIL, high GRIm/low TIL, and high GRIm/high TIL.

Baseline clinicopathologic parameters, including tumor stage, grade, and histologic subtype, showed no significant intergroup differences (all *p* > 0.05).

Median disease-free survival (DFS) was 28 months in the low GRIm/high TIL group, 17 months in the low GRIm/low TIL group, 9 months in the high GRIm/low TIL group, and 12 months in the high GRIm/high TIL group (log-rank χ^2^ = 10.830, *p* = 0.013).

**Figure 4:**
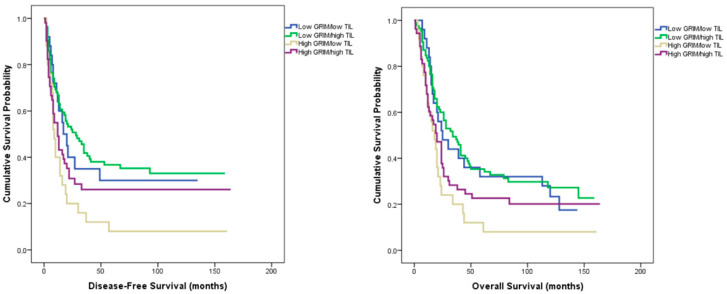
Kaplan–Meier curves for disease-free survival (**left**) and overall survival (**right**) according to combined GRIm–TIL classification.

The corresponding overall survival (OS) values were 34, 25, 18, and 20 months, respectively (log-rank χ^2^ = 10.143, *p* = 0.017) (Figure 4).

In multivariate Cox regression analysis including conventional prognostic parameters (T and N stage, grade, lymphovascular and perineural invasion, and tumor–stroma ratio), the combined GRIm–TIL classification showed a borderline association with both DFS (HR = 1.16, 95% CI 0.97–1.38, *p* = 0.099) and OS (HR = 1.14, 95% CI 0.98–1.34, *p* = 0.115).

## 4. Discussion

To the best of our knowledge, this is the first study to assess the prognostic significance of the GRIm score—a systemic inflammatory marker—in conjunction with pathological features of the tumor microenvironment. Classification by GRIm score revealed no significant baseline differences between low- and high-score groups with respect to age, sex, tumor location, histologic subtype, or grade, indicating a well-balanced cohort. Patients with a low GRIm score demonstrated significantly longer disease-free (24 vs. 12 months, *p* = 0.004) and overall survival (32 vs. 19 months, *p* = 0.006) than those with a high score. Multivariate analysis confirmed the GRIm score as an independent predictor of recurrence (*p* = 0.035) and overall survival (*p* = 0.044). When integrated with pathological variables, prognostic discrimination further improved: the longest survival times were observed in the low GRIm/PD-L1–positive (DFS = 35 months, OS = 39 months; *p* = 0.024, 0.029) and low GRIm/high TIL groups (DFS = 28 months, OS = 34 months; *p* = 0.013, 0.017). The strongest stratification was achieved with the GRIm–TSR model, where patients with low GRIm and low TSR exhibited the most favorable outcomes (DFS = 35 months, OS = 45 months; *p* = 0.014, 0.001), retaining independent prognostic significance in multivariate analysis.

Cancer-related inflammation plays a central role in tumor initiation, progression, and clinical outcomes by shaping both the tumor microenvironment and the systemic host response [8]. Accordingly, increasing attention has been directed toward identifying easily accessible inflammatory and nutritional biomarkers that reflect this systemic response and may improve prognostic stratification beyond conventional clinicopathologic factors. In this context, a growing body of literature has explored biomarkers reflecting systemic inflammation, immune dysregulation, and metabolic status, including C-reactive protein–based indices, neutrophil-to-lymphocyte ratio, and composite inflammation-based scores [23]. More recently, enzymes involved in inflammatory and metabolic pathways have attracted interest as indirect markers of systemic inflammatory burden. Among these, butyrylcholinesterase—a liver-synthesized enzyme that decreases in states of systemic inflammation and malnutrition—has emerged as a potential biomarker reflecting the systemic host response [24]. Although butyrylcholinesterase has been reported to correlate with postoperative infectious complications in colorectal surgery, these findings are widely considered to reflect the underlying systemic inflammatory and metabolic stress, thereby extending its relevance beyond purely procedure-specific outcomes and supporting its role as a marker of global inflammatory and metabolic stress [25]. Importantly, emerging evidence suggests that cholinesterase activity may also have disease-specific prognostic relevance in gastric cancer. In advanced gastric cancer, reduced serum cholinesterase levels have been independently associated with poorer progression-free and overall survival, as well as diminished response to systemic chemotherapy, suggesting that cholinesterase may reflect not only nutritional status but also tumor-related inflammatory burden and host metabolic reserve in this disease [26]. Similarly, in patients with resectable adenocarcinoma of the gastroesophageal junction, diminished preoperative butyrylcholinesterase levels were shown to predict inferior disease-free and overall survival following neoadjuvant chemotherapy, further supporting its role as a marker of systemic inflammation and tumor aggressiveness rather than a procedure-specific phenomenon [27]. Collectively, these observations support the concept that inflammation-linked biomarkers, even when initially investigated in different clinical settings, may reflect shared biological processes relevant to cancer prognosis and underscore the ongoing need for robust and clinically applicable inflammatory biomarkers in gastric cancer.

The prognostic significance of the GRIm score was first demonstrated by Bigot et al. in phase I immunotherapy trials, where it effectively predicted survival outcomes and optimized patient selection [9]. In that study, high GRIm scores were associated with significantly shorter overall survival (20.4 vs. 68.9 weeks; HR 2.9, 95% CI 1.87–4.64), underscoring the detrimental effects of systemic inflammation and nutritional deficiency. Subsequently, Minami et al. validated the prognostic utility of the GRIm score across various NSCLC settings. Among patients receiving immune checkpoint inhibitors, elevated GRIm scores were predictive of poorer survival (3.2 vs. 19.9 months; HR 3.93, 95% CI 2.04–7.58) [10], and similar findings were observed in chemotherapy-naïve pulmonary adenocarcinoma with wild-type *EGFR* (5.1 vs. 18.4 months; HR 2.20, 95% CI 1.47–3.31) [11]. Beyond metastatic disease settings, the GRIm score—originally introduced as a prognostic tool in advanced cancer—has increasingly been investigated in surgically treated, non-metastatic solid tumors. In resectable solid malignancies such as esophageal squamous cell carcinoma and operable pancreatic adenocarcinoma, retrospective studies have consistently demonstrated that a high GRIm score is associated with significantly worse survival outcomes and remains an independent prognostic factor following curative-intent surgery, underscoring its applicability beyond metastatic disease alone [28,29]. Specifically in resectable gastric cancer, a retrospective analysis conducted by Shi et al. showed that GRIm score–based stratification effectively discriminated prognosis, with patients harboring high GRIm scores experiencing significantly shorter disease-free and overall survival, with multivariable analyses confirming the GRIm score as an independent predictor of recurrence and mortality after radical gastrectomy [30]. Collectively, these findings support the notion that the prognostic relevance of the GRIm score is not confined to metastatic disease, but extends to risk stratification in non-metastatic patients undergoing curative surgical resection. Furthermore, a recent meta-analysis encompassing multiple malignancies confirmed the consistent association between high GRIm scores and inferior survival outcomes [31]. Consistent with these prior observations, our results demonstrated that patients with low GRIm scores achieved significantly longer disease-free survival (24 vs. 12 months, *p* = 0.004) and overall survival (32 vs. 19 months, *p* = 0.006) than those with high scores. Multivariate analysis further established the GRIm score as an independent predictor of recurrence (*p* = 0.035) and overall survival (*p* = 0.044), reinforcing its reproducibility and broad prognostic relevance in resectable gastric adenocarcinoma.

Programmed death-ligand 1 (PD-L1) is an important biomarker that facilitates tumor immune evasion by binding to the PD-1 receptor on T cells and suppressing their activation through its overexpression on tumor cells [32]. Recent evidence suggests that the prognostic impact of PD-L1 expression is context dependent and may vary according to tumor site, disease stage, and immune microenvironment. In a meta-analysis including 3291 patients across 15 studies, Gu et al. demonstrated that PD-L1 overexpression was significantly associated with poorer overall survival in gastric cancer (HR = 1.46, 95% CI 1.08–1.98; *p* = 0.01) and correlated with aggressive pathological features such as lymph-node metastasis, venous invasion, and EBV/MSI positivity [33]. Conversely, Böger et al. reported that PD-L1 positivity was significantly associated with better outcomes and served as an independent favorable prognostic factor in a large Western gastric cancer cohort [34]. These divergent findings highlight that the prognostic role of PD-L1 may depend on molecular and geographical heterogeneity as well as the dynamic interplay between systemic inflammation and the tumor immune microenvironment. In line with these observations, our analysis revealed that patients with PD-L1–positive tumors exhibited longer overall survival than those with PD-L1–negative tumors (median OS = 26 vs. 20 months), although the difference did not reach statistical significance (*p* = 0.167). Notably, in the combined GRIm–PD-L1 analysis, the low-GRIm/PD-L1–positive subgroup demonstrated the most favorable outcomes, with median disease-free and overall survival of 35 and 39 months, respectively (*p* = 0.024 and *p* = 0.029). Collectively, these findings indicate that while PD-L1 alone may not serve as an independent prognostic factor, its integration with systemic inflammatory indices such as the GRIm score may enhance prognostic stratification in surgically treated gastric adenocarcinoma.

The tumor–stroma ratio (TSR) is a simple yet powerful histopathologic biomarker that reflects the biological interaction between tumor cells and their microenvironment. A high stromal component has been repeatedly linked to poorer outcomes in solid tumors. In the multicenter UNITED study of over 1300 stage II–III colon cancer patients, stroma-high tumors had significantly shorter disease-free survival (3-year DFS 70% vs. 83%; *p* < 0.001), and TSR remained an independent predictor after multivariate adjustment [35]. Likewise, a meta-analysis of 14 studies (n = 4238) confirmed that stroma-rich tumors correlated with inferior OS and DFS (pooled HR = 1.89 and 2.10; both *p* < 0.001) [36]. In our cohort, patients with low TSR (≤50%) had longer survival than those with high TSR (>50%), with borderline significance for DFS (*p* = 0.054) and a significant difference for OS (median = 37 vs. 20 months; *p* = 0.001). These findings align with prior evidence linking stroma abundance to tumor progression and immune escape. When TSR was combined with the GRIm score, four prognostic subgroups were defined. The low-GRIm/low-TSR group achieved the longest survival (median DFS = 35 months; OS = 45 months), whereas the high-GRIm/high-TSR group had the poorest outcomes (DFS = 12 months; OS = 19 months; *p* = 0.014 and 0.001, respectively). The GRIm–TSR composite also retained independent prognostic value in multivariate analysis (HR = 1.23; *p* = 0.005). Although TSR alone showed only borderline significance for DFS, its prognostic power increased when integrated with GRIm, highlighting the value of combined systemic and microenvironmental assessment in prognostic modeling.

Tumor-infiltrating lymphocytes (TILs) constitute a key component of the tumor immune microenvironment and are critical mediators of the host antitumor response. Numerous studies have shown that high TIL density, particularly CD8^+^ cytotoxic T-cell infiltration, is associated with improved clinical outcomes and prolonged survival across multiple malignancies, including gastric, breast, and lung cancers [37,38,39,40,41]. In gastric cancer, elevated CD3^+^/CD8^+^ TIL levels have been linked to superior disease-free and overall survival, as demonstrated by Pereira et al. and Thompson et al., while recent meta-analyses confirmed their independent prognostic impact [37,42,43]. Likewise, breast and lung cancer cohorts reported that dense intratumoral CD8^+^ infiltration predicted both better response to neoadjuvant therapy and reduced risk of recurrence [39,40,44]. In our cohort, patients with high TIL density (≥10%) exhibited significantly longer disease-free survival (median 32 vs. 18 months, *p* = 0.041) and overall survival (median 41 vs. 24 months, *p* = 0.016) compared with those with low TIL levels. Furthermore, when combined with the GRIm score, the low-GRIm/high-TIL subgroup achieved the best outcomes (DFS = 28 months; OS = 34 months; *p* = 0.013 and *p* = 0.017, respectively), emphasizing that systemic inflammation and local immune activation jointly determine prognosis. Collectively, these findings reinforce that robust lymphocytic infiltration mitigates the adverse impact of systemic inflammatory burden, and that integrating peripheral and tumor-microenvironmental immune parameters enhances prognostic precision.

The GRIm score alone represents a simple, inexpensive, and readily accessible prognostic indicator derived from routinely available laboratory parameters. Its independent association with both disease-free and overall survival in our cohort underscores its clinical utility as an objective measure of systemic inflammation and nutritional status. Beyond its individual prognostic value, however, our findings indicate that combining the GRIm score with tumor microenvironment–related parameters—such as the tumor–stroma ratio (TSR), PD-L1 expression, and tumor-infiltrating lymphocyte (TIL) density—can meaningfully enhance prognostic discrimination. Although direct statistical comparisons between independent models should be interpreted with caution, the numerical gradient in median DFS supports this integrative approach: 24 months in the low-GRIm group, 19 months in the low-TSR group, and 35 months in the combined low-GRIm/low-TSR subgroup. Similarly, the low-GRIm/PD-L1-positive and low-GRIm/high-TIL combinations yielded the most favorable outcomes, underscoring the complementary interaction between systemic inflammatory status and local immune activity. Together, these patterns suggest that incorporating both systemic and microenvironmental markers captures multiple biological layers of tumor progression—ranging from host inflammatory and nutritional state to stromal remodeling and immune response—providing a more comprehensive reflection of tumor aggressiveness and recurrence risk. Consistent with this integrative perspective, the moderate Harrell’s concordance index observed for disease-free survival indicates that while the GRIm score alone provides limited discriminatory performance, its greatest value may lie in contributing to combined prognostic models that incorporate tumor- and microenvironment-related parameters.

It should also be acknowledged that the majority of patients in this cohort were diagnosed at an advanced T stage, with T3–T4 tumors accounting for the majority of cases, while early-stage (T1) disease was relatively uncommon. This stage distribution is consistent with the well-established epidemiological characteristics of gastric cancer, which is marked by aggressive biological behavior, nonspecific early clinical symptoms, and the absence of population-based screening programs in many regions, thereby resulting in a substantial proportion of patients being diagnosed at more advanced stages [45]. In addition, regional and population-specific factors, including variations in health awareness, inequalities in access to healthcare, and delays in seeking medical attention, may further contribute to later-stage presentation at the time of diagnosis. Importantly, the stage distribution observed in our cohort is in line with previously reported real-world gastric cancer series, suggesting that our findings do not reflect an atypical or artificially selected patient population but rather reinforce their external validity. Although the inclusion of all patients who underwent curative-intent surgery was intended to minimize selection bias, the predominance of advanced-stage disease may nonetheless limit the generalizability of our results to patients with earlier-stage gastric cancer. Nevertheless, this characteristic also highlights the clinical relevance of prognostic stratification tools in higher-risk patient cohorts, where accurate risk assessment is particularly critical. Notably, no statistically significant differences in disease stage distribution were observed between GRIm score categories, and multivariable analyses further confirmed the independent prognostic value of the GRIm score beyond established clinicopathologic variables.

This study has several limitations that should be acknowledged. First, its retrospective design inherently carries the risk of selection and information bias, despite dedicated efforts to ensure data completeness and consistency. Second, upfront resection cases were intentionally selected to allow the assessment of tumor biology and recurrence patterns in the absence of the modifying effects of neoadjuvant therapy; this design reflects a treatment era in which neoadjuvant chemotherapy had not yet been established as a guideline-recommended standard and upfront surgery was commonly practiced. While the absence of neoadjuvant therapy may limit the generalizability of our findings to contemporary treatment paradigms, it also enabled the evaluation of treatment-naïve tumor tissue, thereby facilitating a clearer assessment of intrinsic tumor biology and host–tumor interactions. Third, data on Helicobacter pylori infection status and the presence or classification of atrophic gastritis were not systematically available for this retrospective cohort and therefore could not be incorporated into the analysis. Given the established role of *H. pylori* infection in the pathogenesis and progression of gastric cancer, the absence of these variables should be considered a limitation and may have influenced the biological context of the study population. Finally, the relatively modest sample size—particularly within certain combined subgroups—may have reduced the statistical power to detect more subtle yet clinically meaningful differences.

From a methodological perspective, the multi-parameter GRIm-based combined models represent a biologically coherent approach to integrating host- and tumor-related factors within a unified prognostic framework. The generation of stratified subgroups allows a more refined characterization of heterogeneity among patients with resectable gastric cancer, while maintaining clinical feasibility and cost-effectiveness through reliance on routine laboratory and histopathologic assessments. Rather than aiming to propose a definitive prognostic tool, this analysis highlights that easily obtainable clinical and pathological variables—when interpreted collectively—can yield more informative and biologically grounded prognostic insights. Such integrative approaches may serve as a practical bridge between conventional clinicopathologic staging and molecular profiling, ultimately supporting individualized risk assessment and the tailoring of postoperative surveillance or adjuvant treatment strategies in gastric cancer.

## 5. Conclusions

The present study identifies the Gustave Roussy Immune (GRIm) score as a robust and easily applicable biomarker that independently predicts recurrence and survival in resectable gastric adenocarcinoma. Integrating the GRIm score with microenvironmental parameters—such as PD-L1 expression, tumor-infiltrating lymphocyte (TIL) density, and tumor–stroma ratio (TSR)—provides improved prognostic discrimination by jointly reflecting systemic inflammation, host nutritional status, and stromal–immune dynamics. This integrative model offers a biologically coherent and clinically feasible framework for individualized risk stratification and postoperative management. Prospective, multicenter validation studies are warranted to confirm these findings and to explore the potential role of GRIm-based stratification in guiding immunotherapy decisions in gastric cancer.

## Figures and Tables

**Table 1:** Baseline Clinicopathological Features of the Study Population.

Variable	n (%)
Sex	Male 136 (72.3) Female 52 (27.7)
Histologic type	Tubular 85 (45.2) Poorly cohesive 42 (22.3) Mixed 38 (20.2) Papillary 12 (6.4) Mucinous 7 (3.7) Micropapillary 4 (2.1)
Lymphatic invasion	Present 160 (85.1) Absent 28 (14.9)
Perineural invasion	Present 124 (66.0) Absent 64 (34.0)
Nodal stage	N0 45 (23.9) N1 48 (25.5) N2 28 (14.9) N3 67 (35.6)
T stage	T1 12 (6.4) T2 23 (12.2) T3 50 (26.6) T4 103 (54.8)
Tumor location	Corpus 54 (28.7) Antrum 49 (26.1) Diffuse 45 (23.9) Cardia 27 (14.4) Fundus 8 (4.3) Pylorus 5 (2.7)
Tumor stroma ratio (TSR)	Low (≤50%) 107 (56.9) High (>50%) 81 (43.1)
PD-L1 CPS ≥ 1	Positive 93 (49.5) Negative 95 (50.5)
TIL ≥ 10%	<10% 50 (26.6) ≥10% 138 (73.4)

Abbreviations: CPS, combined positive score; TIL, tumor-infiltrating lymphocytes; TSR, tumor–stroma ratio.

**Table 2:** Univariate and Multivariate Cox Regression Analysis for Disease-Free Survival (DFS).

Variable	Univariate HR (95% CI)	*p*-Value	Multivariate HR (95% CI)	*p*-Value
Gender	1.04 (0.69–1.56)	0.855	–	–
Age	0.99 (0.98–1.01)	0.309	–	–
PD-L1 CPS ≥ 1	0.82 (0.58–1.15)	0.251	–	–
Histologic type	1.03 (0.91–1.16)	0.659	–	–
Tumor grade	0.83 (0.64–1.07)	0.143	–	–
Lymphatic invasion	1.73 (1.02–2.92)	0.042	0.94 (0.51–1.75)	0.846
Perineural invasion	1.19 (0.83–1.72)	0.351	0.60 (0.39–0.93)	0.022
T stage	1.51 (1.22–1.87)	<0.001	1.52 (1.19–1.93)	0.001
Nodal stage	1.40 (1.20–1.62)	<0.001	1.35 (1.12–1.63)	0.002
Tumor stroma ratio	1.40 (0.99–1.98)	0.061	1.08 (0.74–1.58)	0.687
TIL (PTL ≥ 10%)	0.75 (0.52–1.09)	0.134	–	–
GRIm category (high)	1.63 (1.15–2.31)	0.006	1.47 (1.03–2.11)	0.035

Note: HR = hazard ratio; CI = confidence interval; TIL = tumor-infiltrating lymphocyte; PTL = peritumoral lymphocyte; GRIm = Gustave Roussy Immune score; TSR = tumor-stroma ratio. Multivariate model includes GRIm category, lymphatic invasion, perineural invasion, T stage, nodal stage, and tumor stroma ratio.

**Table 3:** Univariate and Multivariate Cox Regression Analysis for Overall Survival (OS).

Variable	Univariate HR (95% CI)	*p*-Value	Multivariate HR (95% CI)	*p*-Value
Gender	0.91 (0.64–1.31)	0.624	–	–
Age	1.00 (0.98–1.01)	0.962	–	–
Histologic type	1.01 (0.90–1.14)	0.842	–	–
Tumor grade	0.75 (0.59–0.96)	0.024	0.85 (0.66–1.10)	0.211
Lymphatic invasion	1.68 (1.04–2.73)	0.035	0.85 (0.48–1.50)	0.571
Perineural invasion	1.38 (0.97–1.96)	0.070	0.65 (0.43–1.00)	0.049
T stage	1.45 (1.19–1.76)	<0.001	1.32 (1.08–1.61)	0.008
Nodal stage	1.38 (1.20–1.59)	<0.001	1.27 (1.08–1.50)	0.005
PD-L1 CPS ≥ 1	0.80 (0.58–1.10)	0.173	–	–
Tumor stroma ratio	1.74 (1.25–2.42)	0.001	1.31 (0.92–1.85)	0.136
PTL (TIL ≥ 10%)	0.78 (0.54–1.11)	0.163	–	–
GRIm category (high)	1.57 (1.13–2.19)	0.007	1.41 (1.01–1.97)	0.044

Note: HR = hazard ratio; CI = confidence interval; TIL = tumor-infiltrating lymphocyte; PTL = peritumoral lymphocyte; GRIm = Gustave Roussy Immune score; TSR = tumor-stroma ratio. Variables with *p* < 0.10 in univariate analysis were included in multivariate model.

## Data Availability

The data supporting the findings of this study are available within the article. Further inquiries can be directed to the corresponding author. Correspondence and requests for materials should be addressed to Gökhan Öztürk (E-mail: gokymd@gmail.com).
